# Sirtuins and Proteolytic Systems: Implications for Pathogenesis of Synucleinopathies

**DOI:** 10.3390/biom5020735

**Published:** 2015-05-04

**Authors:** Belém Sampaio-Marques, Paula Ludovico

**Affiliations:** 1 Life and Health Sciences Research Institute (ICVS), School of Health Sciences, University of Minho, Braga 4710-057, Portugal; E-Mail: mbmarques@ecsaude.uminho.pt; 2 ICVS/3B’s—PT Government Associate Laboratory, Braga/Guimarães 4710-057, Portugal

**Keywords:** α-synuclein, ubiquitin-proteasome system, chaperone-mediated autophagy, (macro)autophagy, aging, mitochondria, sirtuins

## Abstract

Insoluble and fibrillar forms of α-synuclein are the major components of Lewy bodies, a hallmark of several sporadic and inherited neurodegenerative diseases known as synucleinopathies. α-Synuclein is a natural unfolded and aggregation-prone protein that can be degraded by the ubiquitin-proteasomal system and the lysosomal degradation pathways. α-Synuclein is a target of the main cellular proteolytic systems, but it is also able to alter their function further, contributing to the progression of neurodegeneration. Aging, a major risk for synucleinopathies, is associated with a decrease activity of the proteolytic systems, further aggravating this toxic looping cycle. Here, the current literature on the basic aspects of the routes for α-synuclein clearance, as well as the consequences of the proteolytic systems collapse, will be discussed. Finally, particular focus will be given to the sirtuins’s role on proteostasis regulation, since their modulation emerged as a promising therapeutic strategy to rescue cells from α-synuclein toxicity. The controversial reports on the potential role of sirtuins in the degradation of α-synuclein will be discussed. Connection between sirtuins and proteolytic systems is definitely worth of further studies to increase the knowledge that will allow its proper exploration as new avenue to fight synucleinopathies.

## 1. Introduction

α-Synuclein (α-syn) is a presynaptic neuronal protein, with 140 amino acids, that was originally identified in association with synaptic vesicles in the presynaptic nerve terminal [1]. α-Syn is ubiquitously expressed in the brain, but it is also present in high amounts in other tissues, including red blood cells [2]. α-Syn’s normal function is still poorly understood, but it is known to be involved in vesicular trafficking, in the regulation of dopamine homeostasis/neurotransmission and in synaptic function/plasticity (reviewed in [2]). α-Syn also appears to influence, when present in low levels, the amount of SNARE complex at the nerve terminal either directly, as a chaperone, or indirectly through other effects on the synaptic vesicle cycle [3]. α-Syn is a major component of Lewy bodies (LB), the hallmark protein inclusions made up of insoluble and fibrillar forms of α-syn [4], implicated in the pathology of several neurodegenerative diseases known as synucleinopathies, including Parkinson disease (PD), dementia with Lewy bodies (DLB) and multiple system atrophy (MSA). Point mutations in the α-syn codifying gene, SNCA, such as A30P [5], A53T [6], E46K [7], G51D [8] and H50Q [9], and genomic multiplications of the SNCA wild type have been identified in families with autosomal-dominant forms of PD [10], indicating that even slight alterations in the α-syn levels could be a risk factor [11]. The mechanism of α-syn aggregation has been largely explored in different studies aiming to identify the toxic species responsible for neuronal dysfunction and death. Multiple studies *in vivo* and *in vitro* PD models showed a strong correlation between α-syn oligomers and neuronal toxicity (reviewed in [2,12]), sustaining that pre-fibrillar, soluble oligomeric α-syn forms are the most toxic α-syn species with a prominent pathogenic role in PD. More recently, it has been shown that α-syn, initially thought to be an exclusive intracellular protein, is secreted to extracellular milieu contributing for the spread of α-syn toxicity and progression of neurodegeneration [13,14,15]. This prion-like mechanism of α-syn spread has been associated with secretion of membrane vesicles, identified as exosomes [16,17]. Both α-syn monomers and toxic oligomers were found to be secreted by non-classical exocytic or endocytic pathways [2,18].

Cells have evolved a protein quality control system to face the danger posed by misfolded proteins, as α-syn monomers and toxic oligomers, and to keep proteostasis [19,20]. These protein quality control mechanisms comprise protein surveillance by molecular chaperones and the function of the two main protein degradation systems, the ubiquitin–proteasome system (UPS) and lysosome-mediated degradation [21,22,23,24,25,26,27,28,29]. Protein quality control mechanisms not only ensure that potential toxic species are detected and eliminated, preventing the accumulation of aberrant forms of proteins and reducing the risks of proteotoxicity, but are also intimately linked to the regulation of the early secretory pathway and protein regulated secretion, particularly lysosome-mediated degradation [30]. Several studies point out α-syn not only as a target of protein quality control mechanisms but also as capable of inhibiting and alter their function further contributing to the progression of neurodegeneration. This vicious cycle is even aggravated if one considered that the activity of the protein quality control mechanisms decreases during aging, a major risk factor for neurodegeneration. Clearly, a better understanding of α-syn proteostasis is aimed and synucleinopathies treatment would greatly benefit from it.

In this review, we will first discuss some basic aspects of the routes for α-syn clearance. Subsequently, we will address the consequences of the proteolytic systems collapse, a major contributor to the pathogenesis of neurodegenerative diseases that could be a primary cause for α-syn accumulation but also a secondary consequence of α-syn aggregates accumulation. Finally, the role of sirtuins on the proteostasis regulation and control will be reviewed as a promising window of opportunity for therapeutic intervention in PD based on proteostasis strategies that are likely to be of value for synucleinopathies.

## 2. Routes for Alpha-Synuclein Clearance

Protein homeostasis is maintained by a balanced equilibrium between a set of interacting cellular pathways that control protein synthesis, folding, trafficking, disaggregation and degradation [31]. As mentioned above, to maintain proteostasis, cells possess protein quality control mechanisms that promote proteolysis in a coordinated fashion, as the UPS or the lysosome/vacuole system [32]. The UPS is essentially the primary route for degradation of short-lived proteins and contributes to the clearance of defective proteins. Protein degradation via the UPS is a highly complex and tightly regulated process that involves a multi-step ubiquitination cascade [33]. In contrast, autophagy/lysosomal is the preferential pathway for degrading long-lived proteins and organelles, contributing equally to the degradation of defective proteins. The term “autophagy” embraces several different mechanisms involved in the lysosomal degradation of cellular components that depend on the type of cargo and the cellular conditions, namely microautophagy, macroautophagy and chaperone-mediated autophagy (CMA) [34]. Microautophagy involves the direct invagination and subsequently engulfment by lysosomes of small pieces of cytoplasm, while macroautophagy (hereafter called autophagy) is characterized by the formation of a double-membrane-bound vacuole, known as autophagosome, which ultimately fuses with the lysosome/vacuole compartment for the degradation of the sequestered cytoplasmic components. In contrast, CMA allows the selective degradation of soluble intracellular proteins by their direct uptake into lysosomes [35]. This process is dependent on the protein chaperone heat­shock cognate 70 (Hsc70) that recognizes the KFREQ motif in the targeted protein and promotes its binding to the lysosomal surface receptor LAMP-2A [36].

**Figure 1:**
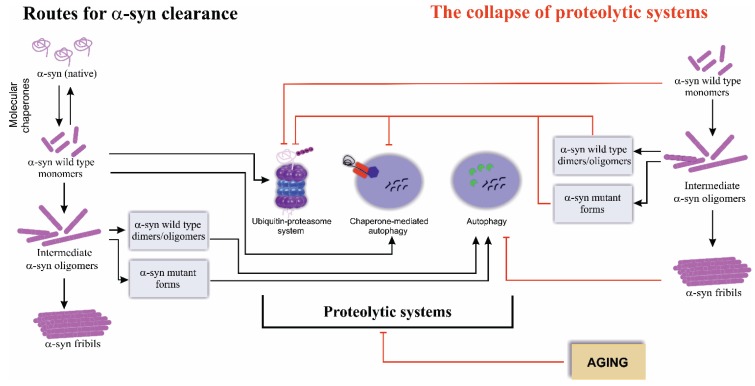
The routes for α-synuclein (α-syn) clearance. α-Syn exists as a soluble monomer that can undergo misfolding into toxic species, dimers and intermediate oligomers, which can further form superfibriliar aggregates. The clearance of different α-syn species occurs via two main proteolytic systems, ubiquitin-proteasome system (UPS) and the lysosome-mediated degradation (particularly chaperone-mediated autophagy (CMA) and macroautophagy (referred as autophagy)). UPS and CMA are the main degradation routes of soluble wild type α-syn, while autophagy is the preferred clearance pathway for intermediate oligomers or mutant α-syn forms. α-Syn levels, mutations and post-translational modifications, impact on the proteolytic systems function leading to their collapse. Aging is also a factor associated with the impairment of the proteolytic systems function, which predispose the cells to the accumulation of α-syn toxic species. See the text for details.

Studies performed *in vitro* showed that, depending on the conformational state, clearance of α-syn can occur both by UPS as well as by lysosome-mediated degradation pathways [37,38] (Figure 1). Multiple evidence showing that (i) LB are immunoreactive for ubiquitin [4,39,40,41,42], p62 [43,44,45], LC3 [46,47], LAMP-2A [46,47] and components of the proteasome [48,49]; (ii) α-syn is present in lysosomes *in vivo* [50,51]; and (iii) genetic alterations of genes codifying proteins involved in proteolytic systems such as Parkin and PINK1 are causative of familial PD [52,53,54,55], further highlights the relevance of clearance routes for α-syn pathology.

Although it has been proposed that under physiological conditions UPS is the preferred pathway for α-syn clearance and that lysosome-mediated degradation pathways assume relevance under pathologic conditions [56,57], the factors that dictate which would be the preferred α-syn clearance route remains puzzling. Apparently, ubiquitination could have a role on the cellular decision of the α-syn clearance route. *In vivo* and *in vitro* studies have showed that α-syn is monoubiquitinated by the E3 ubiquitin-ligase SIAH [58,59,60] and deubiquitined by USP9X [61], both enzymes determining whether α-syn is degraded by UPS. Importantly, the co-chaperone Hsp70 (heat shock protein 70)-interacting protein CHIP connects protein surveillance by chaperones to the ubiquitin dependent degradation of oligomeric α-syn species [62,63]. Intriguingly, CHIP also functions as a switch between α-syn degradation by UPS and lysosome-mediated degradation [62,63]. Actually, lysosome-mediated degradation pathways also have a role on α-syn clearance, as an *in vitro* approach using isolate intact lysosomes showed that purified α-syn wild type protein is translocated into and degraded by lysosome [38]. α-Syn contains the CMA-targeting motif in its amino acid sequence [38] allowing its translocation to lysosomes and clearance via CMA. Cuervo and collaborators, using cell line systems, showed that only wild type α-syn is a CMA substrate, whereas the A30P and A53T α-syn mutant forms are able to strongly bind to LAMP-2A, but are not internalized and degraded, acting as inhibitors of CMA degradation of other substrates [38]. Consistently, the down-regulation of LAMP-2A expression or the expression of α-syn forms lacking the CMA recognition motif resulted in a drastic reduction of α-syn association with the lysosomal membrane and translocation into the lysosome, corroborating that CMA is an important pathway for normal α-syn turnover [64]. Consistently, in human derived dopaminergic cell cultures, it was also showed that wild type α-syn is preferentially degraded via CMA rather than UPS or autophagy [46]. Data obtained on animal models (toxin-based and transgenic mouse model overexpressing wild type α-syn) also pinpoints the relevance of CMA for α-syn degradation, as α-syn was found to co-localize with Hsc70 that, together with LAMP-2A, is up-regulated [65]. In contrast, these same CMA mediators, LAMP-2A and Hsc70, were observed significantly reduced in the brain of PD patients [46] suggesting that α-syn degradation by CMA could be compromised. Importantly, LAMP-2A and Hsc70 genes were found to be down-regulated by a miRNA-mediated process relevant for α-syn pathology as discussed further below [66].

Altogether, the data collected suggests that UPS and CMA are the main degradation routes of soluble α-syn, essentially wild type α-syn (Figure 1). Apparently, although not fully understood, both processes possess distinct roles for α-syn degradation [67]. Nevertheless, accumulating evidence suggests that, under certain conditions such as the expression of α-syn mutant forms or the formation of large aggregates, the UPS and CMA are inhibited and autophagy might work as a default pathway (Figure 1). Ultrastructural examination of postmortem brains of PD patients revealed the presence of intraneuronal autophagic vacuoles (AVs), suggesting a role for autophagy in PD disease [68]. In addition, in a PC12 cell model expressing A30P and A53T α-syn mutant forms, a blockage of CMA and a consequent autophagy activation was observed [38], which is also detected when a proteasome inhibitor, MG132, is used [69]. Consistently, in cortical neurons cultures and SH-SY5Y cells, the expression of A53T mutant α-syn also impacts on CMA function and led to the activation of autophagy [70]. Despite these observations supporting autophagic degradation of α-syn, it is also known that deubiquitinated α-syn is mostly degraded by autophagy in a less efficient way when compared to UPS degradation of monoubiquitinated α-syn [61]. Interestingly, data obtained in different cell models including the data generated in our lab in yeast cells expressing α-syn showed a prolonged and sustained unfolded protein response (UPR) [71,72,73] that could also underlie the observed enhancement of lysosomal degradation pathways.

An important aspect to take into consideration, and often disregarded, when addressing α-syn clearance is the impact that the different oligomerization and fibrillation status of α-syn might have on its clearance and therefore on the degradation systems. The oligomerization and fibrillation of α-syn is particularly sensitive, among others, to pH, temperature, protein concentration, point mutations (A30P, A53T and H50Q α-syn variants favor the formation of toxic oligomeric intermediates [74,75] while the E46K mutation increases amyloid fibril formation [76]) and post-translational modifications [77]. Different α-syn post-translational modifications affecting aggregation properties of α-syn, and thus its clearance, have been observed in LB of PD brains, such as nitration of tyrosines, oxidation of methionines, ubiquitination, nitration, phosphorylation or SUMOylation [77]. Curiously, although 20S proteasome, the catalytic subunit, is able to degrade nitrated monomeric α-syn, it occurs at a slower rate compared with wild type α-syn [78], suggesting that post-translational modification of α-syn by nitration can promote its accumulation [78]. Oxidation, as well as nitration, slightly inhibits α-syn degradation by CMA, while phosphorylation and exposure to dopamine almost completely prevent α-syn degradation by CMA [79]. α-Syn phosphorylation on S129 was also shown to alter the ability of yeast cells to clear α-syn inclusions [80]. Phosphorylated, ubiquitinated, nitrated and oxidized forms of α-syn have been identified in cytosolic aggregates in experimental models and brains from PD patients, suggesting an inhibition of CMA pathway [81,82,83,84,85]. Therefore, numerous findings indicate that various α-syn post-translational modifications impact on the ability of the α-syn, and other substrates, to be degraded via UPS and CMA, the two major degradative pathways involved in α-syn clearance.

The routes for the clearance of the different assembled α-syn species (monomeric, oligomeric or fibrillary) are still controversial in spite of the intense investigations efforts that have been made. Several studies have shown that small and soluble oligomers of α-syn are preferentially degraded by the UPS, while the large oligomeric forms are cleared by autophagy [86], suggesting that in conditions of endogenous and increased α-syn protein burden the UPS is the clearance route, whereas under pathologic context, elevated α-syn levels or presence of oligomeric species, lysosome-mediated degradation is the driven pathway [56]. Among the two main lysosome-mediated degradation pathways, it has also been demonstrated that CMA degrades wild type α-syn monomers and dimers, but not oligomers or α-syn mutant forms that are substrates for autophagy degradation [31].

Summing up, it is clear that protein quality control mechanisms, both UPS and lysosome-mediated degradation pathways, are crucial for the clearance of the different α-syn species. Nevertheless, abnormal α-syn degradation by impairment of those mechanisms might have both beneficial and detrimental effects in synucleinopathies pathology. This anachronism is most probably explained by the fact that the decision in α-syn clearance and the selected route is likely dependent on the α-syn levels, assembly state (monomeric, oligomeric or fibrillary) and post-translational modifications as well as on the cell type, and the stage of the pathogenic process. Thus, the role of α-syn clearance on PD pathogenesis remains highly controversial and complex due to the looping cascade involving α-syn and proteolytic systems. In the next section, the consequences of proteolytic systems collapse will be discussed in the context of α-syn pathology.

## 3. The Collapse of Proteolytic Systems

Impaired protein degradation and protein aggregation are phenomena closely associated with mitochondrial dysfunction and common events occurring during aging, a major risk factor for neurodegeneration (Figure 1). These processes likely overlap and interact contributing for the pathogenic mechanisms of α-syn [87]. Whether the impairment of the protein degradation is a primary cause for the accumulation of aggregated α-syn species, or in contrast, deregulation of proteolytic systems occurs as a consequence of the presence of aberrant α-syn species is still a hot topic on synucleinopathies pathogenesis. Several studies strongly support an interaction between α-syn and the proteolytic systems, leading to the failure of α-syn degradation and its accumulation [31,57,88]. UPS has been consistently described as impaired in synucleinopathies [89]. In a rat model expressing the human mutant A30P α-syn, the 20S proteasome levels were found significantly decreased [48]. Similar results were obtained with PC12 cells overexpressing A53T mutant human α-syn [90] and with toxin-based PD animal models [91,92] showing a marked decrease of proteasome activity and thus a disruption of UPS. Additional accumulated evidence also point out that overexpression of mutant or wild type α-syn variants, particularly soluble oligomeric and aggregated conformations, inhibits the activity of the 20S/26S proteasome [90,93,94]. A significant decrease on both proteosomal activity and of proteasome subunits protein levels was also detected in dopaminergic neurons in the substantia nigra pars compacta (SNc) of sporadic PD patients [95]. These findings point to inadequate UPS activity associated with accumulation of α-syn in PD, implicating proteasomal dysfunction in PD pathogenesis [95,96,97]. It is hypothesized that proteasome inhibition might be due to a direct binding of α-syn to 19S proteasome or to 20S proteasome [93,98,99]. In an attempt to rationalize these observations, it is proposed that translocation of α-syn into the interior of the 20S cylinder results in the binding of α-syn to one or more of the catalytic β-subunits, which simultaneously will interfere with the active site decreasing the overall proteolytic activity of the proteasome [93,98,99]. Alternatively, oligomeric α-syn can directly bind to a subunit of the 19S complex inhibiting substrate recognition or gate opening [17,31]. In summary, a reciprocal crosstalk between α-syn and proteasome function apparently occurs, suggesting a self-perpetuating process, in which α-syn impacts on the UPS activity, which can in turn lead to further α-syn accumulation.

As mentioned above, degradation of several α-syn species also occurs through the lysosome-mediated pathways, particularly CMA, thereby, it has been suggested that impairment of CMA and broadly of lysosome function, might contribute to PD pathogenesis [46,100]. This is supported by the observation that SNc of PD patients present a reduction in the LAMP-2A levels, as well as other lysosomal components, such as LAMP1, cathepsin D and Hsc70 [47,48]. The α-syn mutant forms, A30P and A53T, are also responsible for CMA inhibition as they have the ability to strongly bind to the LAMP-2A receptor [38,70]. CMA dysfunction can be important *in vivo* as accumulation of the CMA substrates such as α-syn and MEF2D, a pro-survival transcription factor, was also observed in a toxin PD model [101]. Consistently, experimental up-regulation of CMA, through overexpression of LAMP-2A has been proven to be beneficial in different cellular systems [31,102]. Nevertheless, it has been shown that the increased α-syn levels per se do not account for the observed reduction of LAMP-2A and Hsc70 protein levels in SNc and amygdala of PD brains [46]. Instead, a major factor influencing those observations was experimentally shown to be the increase in specific miRNAs that down-regulate target LAMP-2A and Hsc70 expression and compromise α-syn degradation [66]. In accordance, the miRNA *MIR128* was found to modulate α-syn-mediated toxicity, dependent of TFEB (transcription factor EB) function. TFEB is a master regulator of lysosome-mediated degradation that is trapped by high α-syn levels preventing it translocation into the nucleus and impairing the induction of the lysosome-mediated degradation pathways [103,104].

Accumulating evidence obtained in different PD models suggests that not only CMA is inhibited in the course of PD but that a more generalized lysosomal dysfunction, suggestive of CMA impairment and excessive induction or failure of autophagy completion occurs [105] (Figure 1). In fact, it was demonstrated that CMA collapse is followed by a compensatory up-regulation of autophagy, supporting a potential crosstalk between the two major lysosome-mediated degradation processes [70,106]. Although, CMA and autophagy are not redundant pathways, compensatory activation of autophagy, in basal conditions, preserves homeostasis in cells with impaired CMA [106]. Equally, it is also established that in response to autophagy blockage, cells might up-regulate CMA [107]. Although autophagy lacks the selectivity that characterizes CMA [108,109,110] and cannot replace CMA under stress conditions [106], the crosstalk between these pathways is of particular interest in the α-syn toxicity context [38,79]. Furthermore, it has been proposed that autophagy induction might also be a response to UPS inhibition. CHIP and USP9X, as referred above, are two promise candidates as regulators of the decision of α-syn degradation by UPS or autophagy [61,62].

The major clearance routes for α-syn degradation, UPS, CMA and autophagy, are therefore, interconnected to each other and blockage of one might up-regulate the others [64,106,107]. Given the primary role of UPS and CMA on α-syn degradation and their impairment in the course of pathology, autophagy induction appears to be a final route to avoid proteolysis collapse. Analysis of the brain of patients with DLB revealed a substantial increase in the autophagy markers LC3-II and beclin1, suggesting that autophagy is stimulated to clear aberrant α-syn inclusions [111]. Nevertheless, the increased autophagy levels are not enough or at least do not promote the α-syn complete clearance, as recapitulated in a transgenic mouse model overexpressing A53T mutant variant, suggesting that autophagy could also be inhibited as happens with UPS and CMA [111]. Curiously, an increase of α-syn levels was shown to result in a decrease of omegasome formation (a portion from the endoplasmic reticulum that is associated with the autophagosome biogenesis), of LC3-II conversion and accumulation of autophagy subtracts in a mouse model and SKNSH cells overexpressing α-syn wild type [112]. Thus, apparently, α-syn is also able to inhibit autophagy at a very early stage of autophagosome formation that has been proposed to be mediated by α-syn impact on the function of Rab1, a GTPase regulating many steps of membrane traffic, including vesicle formation and vesicle movement [113].

Autophagy induction, in spite of the process completion, has also been associated with the promotion of cell death and increased α-syn toxicity in cellular systems, animal models and postmortem PD brains [68,109]. Xilouri and collaborates also demonstrated that the expression of wild type or the mutant A53T α-syn, in SH-SY5Y and PC12 cell lines, resulted in a detrimental up-regulation of autophagy, since its pharmacological or molecular inhibition was protective [70]. The presence of increased numbers of AVs on the brain of PD patients is indicative of excessive induction or failure of autophagy completion and CMA impairment [68], suggesting a generalized lysosomal dysfunction. Importantly, impairment of lysosome-mediated degradation pathways has been associated with the release and presence of extracellular α-syn by *in vitro* assays [114].

The link between synucleinopathies and Gaucher disease (GD), a rare autosomal recessive lysosomal storage disorder associated with mutations in the gene for the glucocerebrosidase (GBA), also pinpoints the crucial role of lysosomal degradation in α-syn clearance. Heterozygous *GBA1* mutations are found in a high frequency in PD patients [115,116] and thus are considered to be the most common genetic risk factor for synucleinopathies [117]. In neuronal cells and in a mouse model of GD, the lack of GBA activity led to defects on proteasomal and autophagic machinery, associated with reductions of LC3-I/II and Atg5/12 [118]. Furthermore, it was recently demonstrated that selective loss of lysosomal glucocerebrosidase early in sporadic PD was directly associated with impaired CMA activity, correlated with reduced LAMP-2A and increased α-syn levels and decreased ceramide [119], as well as accumulation of cytosolic CMA substrate proteins, MEF2D and IκΒα, the nuclear factor (NF) κB inhibitory protein [120].

Accumulating evidence shows that proteostasis declines as a function of time and may provide a reasonable explanation as to why age is a major risk factor for the development of neurodegenerative diseases [121]. The onset of clinical manifestations of synucleinopathies typically occurs at the middle age or later, depending on whether the disease arises from familial or sporadic events. Apparently, neurons might have the ability to maintain toxic proteins in controlled levels for decades. Nevertheless, the age-associated alterations in cellular proteostasis and the compromising of the protein quality control systems might predispose cells to neurotoxicity [122]. A decrease on activity of the UPS with aging due to changes in the gene expression of proteasomal subunits, was observed in both murine muscle [123] and human fibroblasts [124]. Autophagy also declines as a function of age, namely substrate binding and transport to lysosomes, as revealed in rats and human fibroblasts models [125]. Due to the global breakdown of proteostasis with age it is reasonable to hypothesize that proteins could be modified over time. Indeed, cells must cope with the increased burden of misfolded proteins without efficiently means to control protein synthesis, processing, localization, refolding, or degradation. In addition, certain individual combinations of variables or specific genetic backgrounds may render an individual more susceptible to the breakdown of proteostasis, explaining why some individuals succumb to α-syn-associated diseases while others do not.

Interestingly, it was disclosed that old individuals present, in SNc, increased levels of α-syn in comparison with younger individuals [126]. Subsequent studies also reported an increase in the number of α-syn-immunoreactive neurons as a function of age in the SNc [127,128]. Aging is also a factor that could modulate post-translational modification of proteins, as shown by the increased α-syn phosphorylation and nitration in the SNc of aging primate [129].

Aging could also constitute and important risk factor for α-syn toxicity associated with the drastic increase of autophagy. Although increased autophagic activity can mediate α-syn clearance in functionally competent cells, as evidence by several data above described, it might also affect autophagy efficiency and selectivity. Indeed, exacerbated autophagy activity might lead to cell death through surplus consumption of other vital cellular constituents, release of lysosomal hydrolases into the cytosol, or enhanced production of damaging molecules. Our own findings showed that expression of wild type or the mutant A53T α-syn in aged yeast cells promotes a sustained UPR and a drastic increase of autophagy culminating on cell death that is prevented by autophagy inhibition [73]. The observed exacerbated autophagic activity was associated with a drastic increase of mitophagy (mitochondrial autophagy) and loss of mitochondria [73], a vital organelle that is targeted by α-syn and also impaired by aging. Increased mitophagy was associated with mutant α-syn-exerted toxicity in primary neurons, as a cause of mitochondrial dysfunction [73,130]. α-Syn-mediated mitochondrial dysfunction and its interconnection with regulation of proteolytic systems including mitophagy are important concepts on α-syn pathology. Although damaged mitochondria themselves seem to be the proper signal triggering mitophagy [131,132], mitophagy is also indirectly controlled by mitochondrial dynamics (fusion and fission). Results obtained in a transgenic mouse model and SH-SY5Y cells showing that expression of the mutant α-syn resulted in the reduction of mitofusin proteins, Mfn1 and Mfn2, essential for mitochondrial fusion [133], reflect the intricate relationship between mitochondrial dynamics dysfunction and mitophagy. Thereby, α-syn may directly or indirectly damage mitochondria inducing mitophagy. However, the role of mitophagy in α-syn pathogenesis remains unclear: both increased and decreased mitophagy have been proposed to drive toxicity and cellular death under certain circumstances [73,130,134].

In familial forms of PD, two genes, PINK1, encoding the PTEN-induced putative kinase, and Parkin, an E3 ubiquitin ligase, were demonstrated to regulate mitochondrial fission and fusion events and thus the elimination of damaged mitochondria through mitophagy [135,136]. Mitochondrial impairment results in stabilization of PINK1 on damaged mitochondria that promotes Parkin recruitment to the mitochondrial surface, where it ubiquitinates several protein targeting mitochondria for removal by mitophagy [137]. Overexpression of A53T mutant α-syn in cortical neurons was shown to increase Parkin-dependent mitophagy [130] and the mitochondria number in autophagosomes [138]. This phenomenon was partly reverted through mitophagy inhibition, either by general suppression of autophagy or by depletion of Parkin [138]. Consistently, abrogation of Parkin, in double transgenic mice, ameliorated neuropathological and behavioral deficits of α-syn overexpression [139]. In fact, it is hypothesized that α-syn-dependent excessive mitophagy, beyond removing dysfunctional mitochondria, may also eliminate functional mitochondria, thereby decreasing mitochondrial mass, inducing bioenergetics failure and cell death. Supporting this hypothesis, our findings obtained in aged yeast cells heterologous expressing wild type or A53T mutant α-syn showed an exacerbated increase of mitophagy. When α-syn-mediated mitophagy is blocked by deletion of *ATG11* or *ATG32* genes (mitophagy associated genes), α-syn-induced toxicity is abrogated [73]. This data showed that exacerbated mitophagy induced by α-syn has a deleterious effect in aged cells that have a reduced ability to simultaneously up-regulate anabolic processes in order to compensate for the loss of cellular material by increased mitophagy [73]. Simultaneously, the selectivity of various types of autophagy might be lost due to the limited availability of factors/players when mitophagy is up-regulated. Together, the data above presented suggests a pivotal role for mitophagy in α-syn pathology that, apparently, is age dependent.

The successive failure of protein degradation pathways, as a cause or consequence of α-syn toxic species provides a key step in the pathological cascade that leads to neurodegeneration. Aging, a key player on neurodegeneration, precipitates the dysfunction of the protein degradative systems and mitochondria, being a crucial factor that might trigger the beginning of the cellular collapse. Understanding the crosstalk between the proteolytic systems, mitochondrial dysfunction and aging in synucleinopathies, as well as how modulation of these molecular pathways can improve or revert the course of disease, might be a good strategy to fight against the detrimental effects promoted by α-syn.

## 4. The Role of Sirtuins on the Proteostasis Control

Loss of proteostasis associated with a burden and an impairment of the proteolytic pathways is one of the hallmarks of α-syn-induced toxicity. Therefore, modulation of the proteolytic molecular pathways that are deregulated appears as a rational strategy to fight against the harmful effects promoted by α-syn.

Sirtuins are a family of highly conserved NAD^+^ dependent histone deacetylases that have emerged as central players in several biological processes, such as transcription, apoptosis, DNA repair, stress cellular response and energetic metabolism [140]. Mammalian sirtuins family is composed of seven members (SIRT1–SIRT7), being SIRT1 evolutionarily the closest to the yeast Sir2, the founding member of the sirtuins family, first discovered in *Saccharomyces cerevisiae* [140,141]. SIRT1 has been most extensively reviewed in the literature due to its predominant expression in neurons [142]. Furthermore, SIRT1 deacetylates a number of important transcription factors such as peroxisome proliferator activated receptor alpha (PPARa), PPAR gamma coactivator 1 alpha (PGC-1a), NF-κB and the forkhead box, subgroup O (FOXO) family of transcription factors (reviewed in [143]).

The interest in sirtuins, in the context of proteostasis, emerged with the discoveries that sirtuins have the ability to modulate proteostasis, particularly the autophagy degradation pathway, and aging [144]. Analyses of sequence variants within the SIRT1 gene promoter regions in PD patients revealed the existence of three heterozygous sequence variants (g.69644133C>G, g.69644213G>A, and g.69644351G>A), suggesting that polymorphisms may alter the transcription factor sites of SIRT1 gene promoter resulting in decreased SIRT1 levels and increased PD risk [145]. This change on Sirt1 levels could underlie alterations on lysosome degradation processes, which is also supported by observations in the brain of Alzheimer’s disease patients, where a deficiency in SIRT1 leads to hyperacetylation of the phosphorylated tau, resulting in impaired tau degradation by the 26S proteasome [146] and by autophagy [147]. Overexpression of SIRT1 in animal and cell PD models was shown to suppress the formation of α-syn aggregates by the activation of heat shock factor 1 (HSF1), which affects transcription of molecular chaperones such as Hsp70 [143,148]. Overexpression of the SIRT1 homologue also suppresses the formation of α-syn inclusions in *Caenorhabditis elegans* [149]. The promoted effects of SIRT1 are believed to be, at least, partially depended on its ability to induce autophagy, as shown in human cells *in vitro* and in *C. elegans in vivo* [150]. Nevertheless, the promising effects of SIRT1 are not well established and contradictory findings are described. Overexpression of SIRT1 in a PD toxin-based mouse model did not promote neuroprotective effects [151]. Consistently, it was found that in yeast cells deletion of *SIR2* gene, the homologue of mammalian SIRT1, alleviates α-syn toxicity [152] and that this phenomenon, as we showed, is dependent on the Sir2 transcriptionally up-regulation of *ATG8* (homologue of LC3) and *ATG32* (yeast mitophagy-specific gene) culminating in the induction of exacerbate autophagy and mitophagy activities [73], with deleterious effects in aged cells. Therefore, the promising effects of SIRT1 have to be further elucidated and for sure different factors such as post-translational modifications as well as the cell type and the stage of the pathogenic process will influence whether or not SIRT1 will be neuroprotective.

In spite of the discordant data obtained with genetic manipulation of SIRT1, activation of SIRT1 by resveratrol, a natural polyphenol, treatment has been shown to be protective in cell line, worm and mouse models of PD [153,154,155]. Resveratrol protected human neuroblastoma SK-N-BE cells from the toxicity induced by expression of α-syn A30P mutant form by a SIRT1-dependent mechanism as revealed by data obtained with sirtinol, a specific inhibitor of SIRT1 [153]. Although resveratrol has pleiotropic beneficial effects, it is a well-known inducer of autophagy. This was supported by findings showing that knockdown or knockout of SIRT1 prevented the induction of autophagy by resveratrol in human cells as well as by dietary restriction in *C. elegans* [150]. Consistently, it was described that PC12 cell expressing α-syn and treated with resveratrol present increased levels of LC3-II, which is regulated by the SIRT1 activity suggesting that SIRT1-autophagy pathway plays an important role in neuroprotection [155].

Altogether, the accumulated evidence supports a relevant role for SIRT1 in synucleinopathies and definitely, although still controversial, SIRT1 effects on the regulation of autophagy are an important aspect to take into consideration in this complex scenario. Up-to date, and to the best of our knowledge, there is no strong evidence about the impact that SIRT1 might have on other proteolytic pathways besides autophagy. Nevertheless, the effects that SIRT1 has on mitochondria biogenesis could not be disregarded in this context. In fact, as previously mentioned, SIRT1 is a transcriptional activator of PGC-1α (a transcriptional co-regulator that governs mitochondrial biogenesis and activity) that can compensate for neuronal mitochondrial loss due to α-syn expression [156]. Furthermore, dopaminergic SN4741 cells exposed to 1-methyl-4-phenylpyridinium (MPP+) treatment with resveratrol activated PGC-1α, via SIRT1 and enhanced PGC-1α gene transcription with increases in SOD2 (superoxide dismutase 2) and Trx2 (thioredoxin) levels [157]. Thus, it can also be hypothesized that SIRT1 surveillance of mitochondrial functions would counteract the unsustainable loss of mitochondria due to over activation of autophagy. The scenarios where this complex interaction is protective have to be further clarified.

SIRT1 is not the only member of the sirtuins family linked to the modulation of α-syn toxicity. In 2007, a compound shown to function as a SIRT2 inhibitor was found to increase the inclusion size of α-syn aggregates [158]. The SIRT2 inhibitors were shown to rescue cells from α-syn mediated toxicity in a dose-dependent manner and were able to protect dopaminergic neurons from cell death in a Drosophila model of PD [158]. Although the mechanisms responsible for cytoprotective effects of SIRT2 inhibition are still elusive, it is known that SIRT2 inhibitors decrease the number but increase the size of α-synuclein aggregates in a human cell model [158], consistently with the current view that large α-syn aggregates are protective while the oligomeric species are the main toxic α-syn species. Concomitantly, SIRT2 increases protein accumulation in murine cholinergic SN56 cells and human neuroblastoma SH-SY5Y cells in conditions of proteasome inhibition. When overexpressed, SIRT2 has the capacity to inhibit autophagy by decreasing the aggresome formation and increasing the accumulation of LC3-II and p62 proteins, rendering the cells more vulnerable to amyloid beta [159].

Importantly, SIRT2 promotes the deacetylation of Foxo3a, a member of the FoxO (forkhead box O) family of transcription factors, that regulates the expression of genes involved in multiple cellular functions as oxidative stress, apoptosis and autophagy [160,161]. A proposed model based on data produced in a toxin based mouse model of PD predicts that SIRT2 is activated in response to stress causing Foxo3a deacetylation, which leads to increased levels of the pro-apoptotic factor Bim and neuronal death [162]. But not only SIRT2 deacetylates Foxo3a, SIRT1 is also able to do it by modulating its gene transcription and specificity [163]. However, it is still unknown whether acetylation of Foxo3a affects expression of autophagy-related genes. The answer to this conundrum will for sure enlighten the role of sirtuins on proteolytic systems function and α-syn pathology.

Recently, it was demonstrated a noticeable deacetylation of mitochondrial matrix proteins accompanied with decreased levels of SIRT3, the main mitochondrial matrix deacetylase, in the brain of a PD mouse model [164]. Although, this data still needs further investigation, the authors suggested that the mitochondrial matrix deacetylation is due to abnormal lysine acetyltransferase activity, and that the SIRT3 down-regulation represents a compensatory mechanism. Thus, this altered mitochondrial matrix acetylation could be linked with the known mitophagy deficit of PD brains [164].

In combination, the data from several studies evidenced that although modulation of sirtuins appears to be a promising therapeutic strategy, probably due to their impact in aging and autophagy regulation, the precise sirtuins functions are still unclear. Extensive activation of sirtuins leads to deacetylation of several subtracts, histones and non-histone proteins, which consequently will affect several different cellular functions. Thereby, understanding the mechanisms underlying the protective or detrimental role of sirtuins could bring us closer to the identification of promising novel drug targets and for the design of new and more successful therapeutic strategies to fight synucleinopathies.

## 5. Conclusions

The relation between α-syn toxicity and proteostasis particularly proteolytic systems has proven infinitely more complex than ever imagined. Based on a wealth of studies in many different synucleinopathies models, it is now well appreciated that distinct proteolytic pathways are specifically activated in an attempt to degrade diverse α-syn species (conformational, mutated variants and post-translated modified). The progress in the knowledge on the proteolytic systems and α-syn biology/pathology revealed that proteolytic systems could become overload with high α-syn amounts but that various α-syn species could directly/indirectly promote their impairment and inhibition. Further confounding this scenario, aging, a major risk factor for the development of neurodegenerative diseases, is also associated with a decline of protein degradation systems function. The search for molecules able to rescue cells from α-syn toxicity has evidenced sirtuins as promising targets, particularly due to their impact on aging and on autophagy regulation. Nevertheless, similarly to what was found with proteolytic systems, the effects and regulation of sirtuins have proven to be extremely complex. Conflicting data shows opposing roles for sirtuins in the modulation of α-syn toxicity and the lack of knowledge on the mechanisms triggering protective or detrimental effects on synucleinopathies and whether they are mainly dependent of α-syn degradation and/or on mitochondria biogenesis.

Despite the controversial, current concepts on synucleinopathies accept proteolytic systems as a central player on α-syn pathology. Thus, deciphering the mechanisms by which the different proteolytic systems contribute to the elimination or accumulation of α-syn toxic and non-toxic species is therefore of utmost importance and can pave the way for development of new treatment strategies.
